# Beyond Structure: The Role of the Outer Integument in Embryo, Endosperm, and Seed Development in *Annona*

**DOI:** 10.1111/ppl.70538

**Published:** 2025-09-24

**Authors:** María Garcia‐Lezama, José I. Hormaza, Enrique Lopez‐Gomez, Noé Fernandez‐Pozo, Jorge Lora

**Affiliations:** ^1^ Breeding and Developmental Biology of Subtropical Fruit Instituto de Hortofruticultura Subtropical y Mediterranea ‘La Mayora’ (IHSM‐UMA‐CSIC) Málaga Spain

**Keywords:** *Annona*, embryo development, *INNER NO OUTER* (*INO*), nucellus expansion, seed development

## Abstract

In most angiosperms, the embryo develops within maternal tissues and is enclosed by two integuments, which play a crucial role in embryo and seed development. However, while this role has been extensively studied in model eudicots like *Arabidopsis thaliana* , it remains understudied in non‐model plants and largely unexplored in basal and early‐divergent angiosperms. Here, we examine the role of the outer integument in embryo development and seed formation using the unique spontaneous mutant “*Thai Seedless*” of the early‐divergent angiosperm *Annona squamosa* (Annonaceae). This mutant lacks the outer integument due to a deletion in the *INNER NO OUTER* (*INO*) gene, leading to seed abortion. Comparative histological and transcriptomic analyses between the *Annona ino* mutant and wild‐type *Annona* show that, unlike the polar growth observed in the nucellus of the wild‐type genotype, *Annona ino* exhibited lateral nucellus expansion. Gene ontology (GO) enrichment revealed increased expression of reproductive development genes in wild‐type seeds, while *Annona ino* showed altered gene expression patterns associated with cell death. These findings highlight both morphological and transcriptomic differences between *Annona ino* and wild‐type genotypes, demonstrating that the absence of the outer integument disrupts early seed morphology and gene regulation, providing new insights into the genetic and developmental roles of integuments in seed development.

## 1 Introduction

Ovules play a central role in sexual plant reproduction, as they are the precursors of seeds. In angiosperms, ovule morphology is generally conserved, with the nucellus containing the embryo sac (female gametophyte) surrounded by the integuments. The most common type of embryo sac in angiosperms, the *Polygonum* type, consists of seven cells and eight nuclei: an egg cell and two synergids at the micropylar end, a central cell with two nuclei, and three antipodal cells at the chalazal pole (Maheshwari 1950; Mansfield et al. 1991). Most angiosperms have anatropous (inverted toward the placenta) and bitegmic (two integuments) ovules, although variations such as unitegmic (a single integument) or ategmic (no integuments) exist (Endress 2011). In *Arabidopsis thaliana* , the anatropism of mature ovules depends on the asymmetric growth of the outer integument (Robinson‐Beers et al. 1992), a mechanism likely conserved across most angiosperms. The inner or both integuments contribute to forming the micropyle, a narrow canal through which the pollen tubes access the nucellus, extend into the embryo sac, and finally enter the synergid cells. During the double fertilization process of angiosperms, one of the two sperm cells contained in the pollen tube fuses with the egg cell, resulting in the formation of a zygote, and the other fuses with the polar nuclei to form the endosperm. Following fertilization, the integuments develop into the seed coat, a protective outer covering surrounding the plant embryo that is essential for protecting the embryo from mechanical damage and pathogen invasion, ensuring its dehydrated state until germination conditions are favorable (Windsor et al. 2000; Haughn and Chaudhury 2005).

In addition to their structural and protective functions, increasing evidence highlights the role of the integuments in mediating communication between the sporophyte and the female gametophyte. Extensive research has shown that mutants defective in integument development often lead to defective embryo sac formation and reduced fertility (Robinson‐Beers et al. 1992; Wei et al. 2020; Cai et al. 2021; Chen et al. 2023; Qin et al. 2023). Specifically, the asymmetric growth of the outer integument seems to be essential for the proper formation of the female gametophyte.

In *Arabidopsis thaliana* , mutations affecting integument development can severely impact embryo sac formation and fertility. The initiation of the outer integument is regulated by the *INNER NO OUTER* (*INO*) gene, a transcription factor belonging to the YABBY protein family, which is related to polarity determination in lateral organs (Villanueva et al. 1999). Loss‐of‐function *ino* mutants of *Arabidopsis* completely lack the outer integument, while the inner integument seems to develop normally. In these mutants, the formation of the embryo sac is compromised since megagametogenesis fails to proceed (Villanueva et al. 1999). These results suggest that integuments play an essential role in supporting female gametophyte development.

While research in *Arabidopsis* has been fundamental for understanding the genetic mechanisms underlying key processes during female gametophyte and seed development, there is a need for genetic studies in other flowering plants to gain a broader evolutionary perspective of the role of the integuments in embryo development across angiosperms. To fill this gap, studies of mutants with defective integument formation become particularly valuable. One such case is the spontaneous “*Thai seedless*” (*Annona ino*) mutant of *Annona squamosa* , which represents a unique example of stenospermocarpy. This mutant is particularly relevant for ovule development studies due to a mutation in the *INO* gene that results in the absence of the outer integument of the ovule and subsequent seed abortion (Lora et al. 2011).

*Annona squamosa* belongs to the Annonaceae family, one of the largest extant families among early‐divergent angiosperms, comprising approximately 130 genera and 2300 species of trees and shrubs with pantropical distribution (Chatrou et al. 2012). In addition to its evolutionary significance, the Annonaceae family includes several economically important fruit crops, such as cherimoya ( *Annona cherimola* ), sugar apple ( *A. squamosa* ), atemoya hybrid ( *Annona cherimola* × *Annona squamosa* ), and soursop ( *A. muricata* ). These fruits, which were already used as food sources by pre‐Columbian civilizations in South and Central America (Popenoe et al. 1989), are now emerging as commercial crops in various countries with tropical and subtropical climates. However, their market potential remains limited, and the development of seedless varieties could enhance consumer appeal and drive market expansion.

The *Annona ino* mutant exhibits phenotypic effects in the ovules identical to the *A. thaliana* *ino* mutant, as both display orthotropous and unitegmic ovules instead of the anatropous and bitegmic ovules typical of their respective wild‐type genotypes (Gaiser et al. 1995; Baker et al. 1997). However, unlike *A. thaliana ino* mutants, in which the absence of the outer integument disrupts embryo sac formation and prevents fruit development, *Annona ino* mutants still form functional *Polygonum*‐type embryo sacs at anthesis and can produce fruits following pollination (Lora et al. 2011). Thus, *Annona ino* represents a unique mutant case to investigate the evolutionary role of the outer integument in embryo and fruit development.

In this study, we investigate the role of the outer integument in embryo and seed development using the *Annona ino* “*Thai seedless*” mutant. By comparing morphological and gene expression differences between *Annona ino* and wild‐type *Annona* genotypes, we aim to provide new insights into the function of the outer integument in embryo and fruit development.

## 2 Materials and Methods

### 2.1 Plant Material

Adult trees of the *A. squamosa* “*Thai seedless*” mutant (*Annona ino*), the *A. cherimola* cultivar Campas, and the atemoya ( *A. squamosa* × *A. cherimola* hybrid) cultivar Gefner, which served as the wild‐type *Annona* in this study, were used in these experiments. These trees are maintained in a field collection at the Instituto de Hortofruticultura Subtropical y Mediterránea La Mayora (IHSM La Mayora CSIC‐UMA) in Málaga, Spain.

### 2.2 Pollination Procedures

Anthers and pollen of *A. cherimola* were collected from flowers at anther dehiscence and stored at 4°C to use in hand pollination the following day. Flowers of *Annona ino* and wild‐type *Annona* were pollinated at 9:00 h on the first day of the flower cycle.

### 2.3 Histology

To study embryo development, fruitlets from the hand‐pollinated flowers of *Annona ino* and atemoya were collected and fixed at 8, 13, 18, and 22 days after pollination (DAP). The samples were fixed in 4% paraformaldehyde in phosphate‐buffered saline (PBS) at pH 7.3, dehydrated in an acetone series, embedded in Technovit 8100 (Kulzer & Co), and sectioned at 2 μm. Sections were stained with periodic acid‐Schiff's reagent (PAS) and toluidine blue (Sigma‐Aldrich) for general histological observations (Feder and O'Brien 1968). Early‐developing seeds were observed with a Leica DM LB2 microscope.

### 2.4 Nucleic Acid Methods

Total RNA was extracted from pollinated pistils containing early seeds (PS) and pistils without early‐developing seeds (P) of *Annona ino* and wild‐type *Annona*, combining the CTAB DNA extraction protocol (Doyle and Doyle 1987) and the Qiagen RNeasy Plant Mini kit (Qiagen). The P samples were obtained by removing early‐developing seeds from the pistils of young fruits through microdissection with a microscalpel under a stereomicroscope. For each genotype, RNA was extracted from both PS and P samples obtained from three individual fruits at 4 and 8 days after pollination (DAP).

### 2.5 RNA Sequencing

Library preparation (poly‐A enrichment) and mRNA (cDNA) sequencing were conducted at the central sequencing facilities of Novogene Company Ltd. using an Illumina NovaSeq PE150 sequencer, aiming to obtain 30 million read pairs per sample.

### 2.6 Transcriptome Data Processing and Analysis

Raw sequencing reads were evaluated with FastQC v0.11.9 (https://www.bioinformatics.babraham.ac.uk/projects/fastqc) and summarized with MultiQC v1.13a (Ewels et al. 2016). Quality filtering and adapter trimming were performed using Trimmomatic v0.39 (Bolger et al. 2014), with the parameters ILLUMINACLIP:TruSeq3‐PE.fa:2:30:10 LEADING:3 TRAILING:3 SLIDINGWINDOW:4:15 MINLEN:36. The processed reads were aligned to the *Annona cherimola* v102 genome (Talavera et al. 2023) using HISAT2 v2.2.1 (Kim et al. 2019), and the resulting alignments were converted to sorted BAM format with SAMtools v1.16 (Danecek et al. 2021). The gene expressions were quantified at the exon level using featureCounts from the Subread package v2.0.6 (Liao et al. 2014).

Differential gene expression and functional enrichment analyses were conducted using R v4.4.1. DESeq2 v1.46.0 (Love et al. 2014) was employed for normalizing gene expression data and identifying differentially expressed genes (DEGs) based on an adjusted *p* < 0.05. Orthologs between *Annona cherimola* and *Arabidopsis thaliana* were identified using Diamond BLASTp v2.1.9.163 (Buchfink et al. 2015), retaining reciprocal best hits and filtering out matches with a bit‐score below 45. The proteins of *Arabidopsis thaliana* most similar to the DEGs found were utilized for the functional enrichment analysis, which was performed using a custom script (https://github.com/bullones/FunRichR) based on the clusterProfiler package v4.14.6 (Wu et al. 2021).

### 2.7 Early‐Seed Shape

To evaluate the circularity of the nucellus during the early stages of seed development, the circular index (CI) (Schwarz 1980) was calculated using the formula

$$I=4\pi \frac{\text{area}}{{\text{perimeter}}^{2}}$$

This metric quantifies the similarity between a plane figure and a circle, with values ranging from 0 to 1, with 1 corresponding to a perfect circle. This metric provides a useful approximation for evaluating seed shape at early developmental stages. Additionally, the aspect ratio (AR), defined as the ratio between the seed length and width (Bauer et al. 2024), was calculated. To compute the circular index and AR, we measured the area, perimeter, length, and width of the nucellus in 20–30 immature seeds per day at 8, 13, 18, and 22 DAP in *Annona ino* and wild‐type *Annona* (total *n* = 214). These measures allowed us to evaluate changes in seed morphology during embryo development. To determine changes in nucellus cell size and number, the area of the cells near the endosperm was measured in 5 ovules at 22 DAP from *Annona ino* and wild‐type *Annona* (*n* = 10).

Images were captured using a ZEISS SteREO Discovery.V20 microscope equipped with an Axiocam 712 color camera. Measurements were obtained using the ZEISS ZEN 3.10 and ImageJ software. The equality of means for the circularity index and the AR was assessed using an ANOVA, followed by a Tukey test (*p* < 0.05) performed with the RStudio software. For the cell area analysis, a Mann–Whitney test was employed, while a Student's *t*‐test was performed to compare cell numbers.

## 3 Results

### 3.1 Embryo and Endosperm Development

To evaluate the effect of the outer integument on embryo and fruit development, we first examined zygote formation and the subsequent first division of the embryo in *Annona ino* and compared it to atemoya, the hybrid *A. cherimoya × A. squamosa*, which served as the wild‐type *Annona* in this study. At anthesis, wild‐type *Annona* ovules are anatropous, bitegmic, and crassinucellate, with an endostomal micropyle formed by the inner integument, which extends outward beyond the external integument. The mature embryo sac shows the *Polygonum*‐type structure, which was also observed in *Annona ino*, as reported previously (Lora et al. 2011).

**FIGURE 1:**
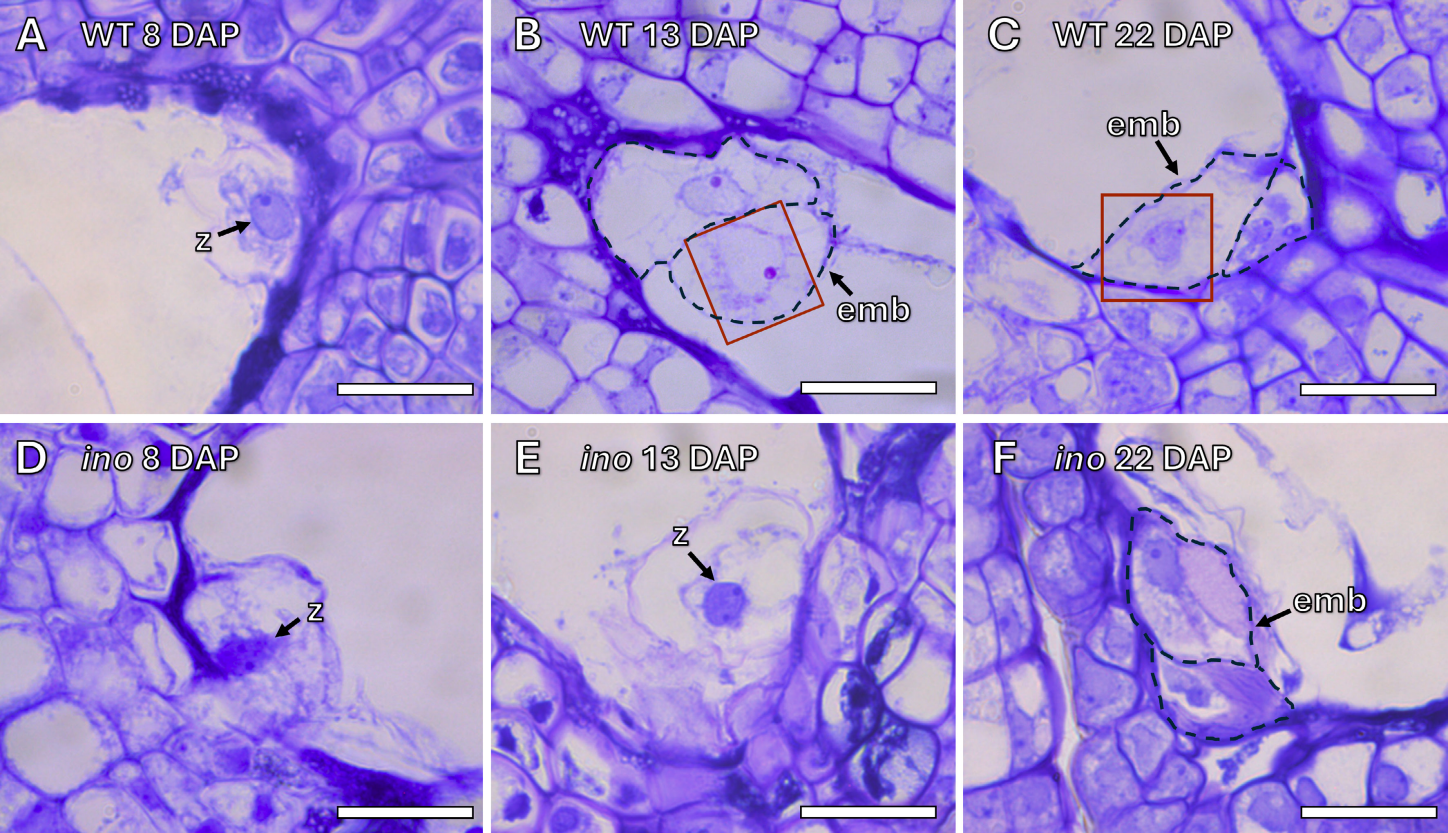
Embryo development in wild‐type *Annona* and *Annona ino*. (A) Zygote at 8 days after pollination (DAP) in wild‐type *Annona*. (B, C) First cellular division forming a two‐celled embryo at 13 DAP (B) and 22 DAP (C) in wild‐type *Annona*. (D, E) Zygote with two prominent nuclei (arrow) at 8 (D) and 13 DAP (E). (F) Two‐celled embryo at 22 DAP in *Annona ino*. Squares around nuclei in some micrographs indicate that the highlighted areas have been copied from adjacent serial sections to create a composite image (adjacent sections in Figure S1). Dashed lines outline the boundaries of the two‐celled embryo. emb, embryo; z, zygote. Sections (2 μm thick) were stained with periodic acid‐Schiff (PAS) reagent and toluidine blue staining. Scale bars: 20 μm.

In wild‐type *Annona*, the zygote was visible at 8 DAP (Figure 1A). The first cellular division, producing a two‐celled embryo, took place 13 DAP (Figures 1B and S1), with the two‐cell embryo still present at 22 DAP (Figures 1C and S1). In *Annona ino*, approximately 50% of pollen tubes reached the base of the ovary and the micropyle (Lora et al. 2019). The zygote was visible at both 8 and 13 DAP, characterized by two prominent nucleoli (Figure 1D,E). However, the first cell division did not occur until 22 DAP, resulting in a two‐celled embryo (Figure 1F). In *Annona ino*, 27.3% (*n* = 22) of early‐developing seeds contained a two‐cell embryo, whereas no embryo could be identified in the remaining 72.7%. In wild‐type *Annona*, 45% (*n* = 33) of early‐developing seeds showed an embryo at 22 DAP.

**FIGURE 2:**
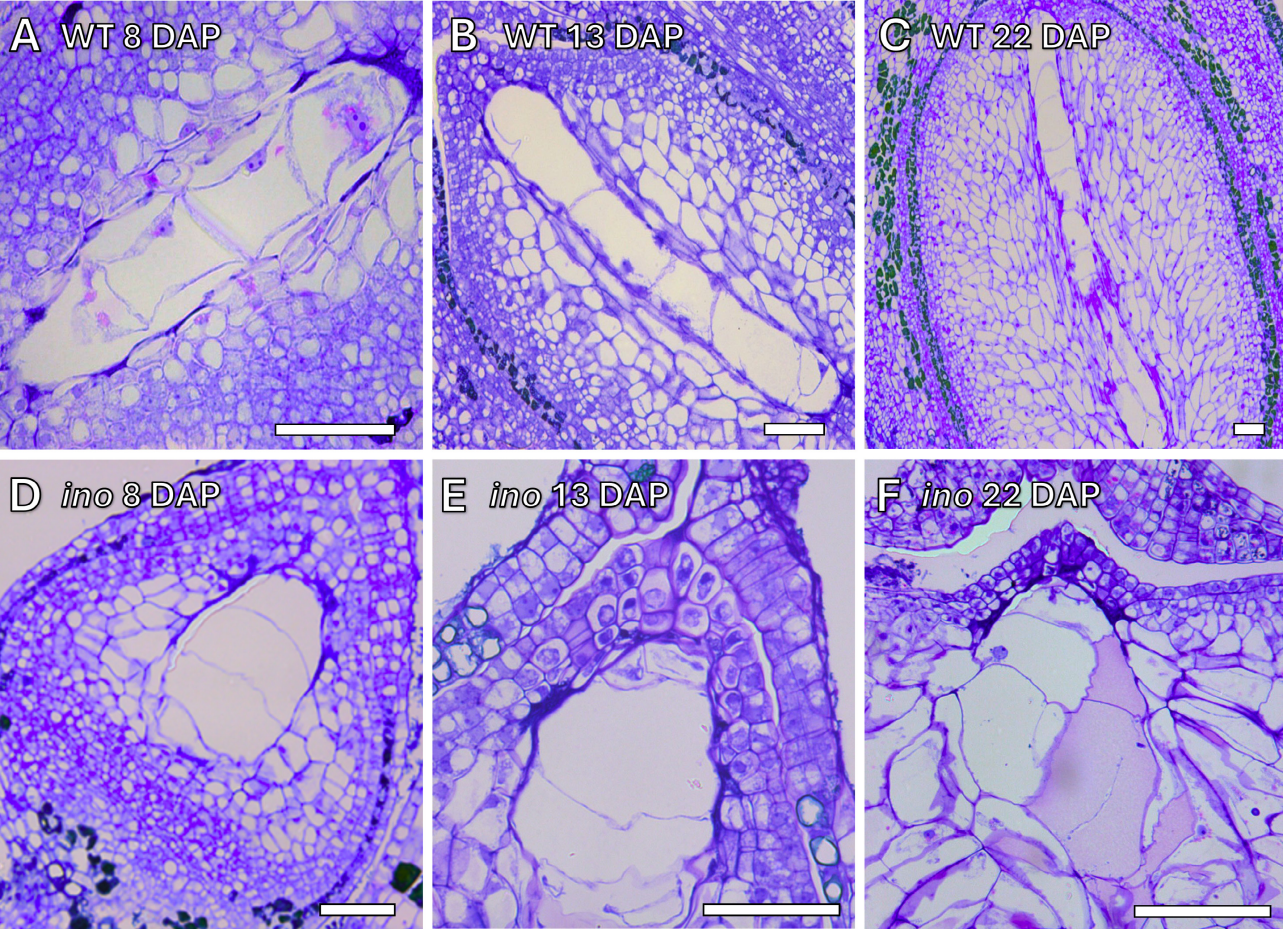
Endosperm development in wild‐type *Annona* and *Annona ino*. (A–C) Cellular endosperm in wild‐type *Annona*, showing (A) 4 cells at 8 days after pollination (DAP), (B) 5–6 cells at 13 DAP, and (C) 9–10 cells at 22 DAP. (D–F) Early‐degenerating endosperm with a maximum of 4 cells observed between 8 and 22 DAP in *Annona ino*. The micropylar (distal) end of the early seed is oriented at the top of the figure, while the chalazal (proximal) end is at the bottom. Sections (2 μm thick) were stained with periodic acid‐Schiff (PAS) reagent and toluidine blue. Scale bars: 50 μm.

While the zygotes remained quiescent, a cellular endosperm with four cells was observed in wild‐type *Annona* at 8 DAP (Figure 2A). By 13 DAP, the endosperm showed 5–6 cells (Figure 2B), and by 22 DAP, it had expanded to 9–10 cells in 100% of the early‐developing seeds (*n* = 51) (Figure 2C). In *Annona ino*, an incipient, early‐degenerating endosperm was identified in 39% of the early‐developing seeds (*n* = 51), with a maximum of 4 cells observed between 8 and 22 DAP (Figure 2D–F), whereas no endosperm was detected in the remaining 61% of the post‐pollination ovules.

### 3.2 Shape of Early‐Stage Developing Seeds

**FIGURE 3:**
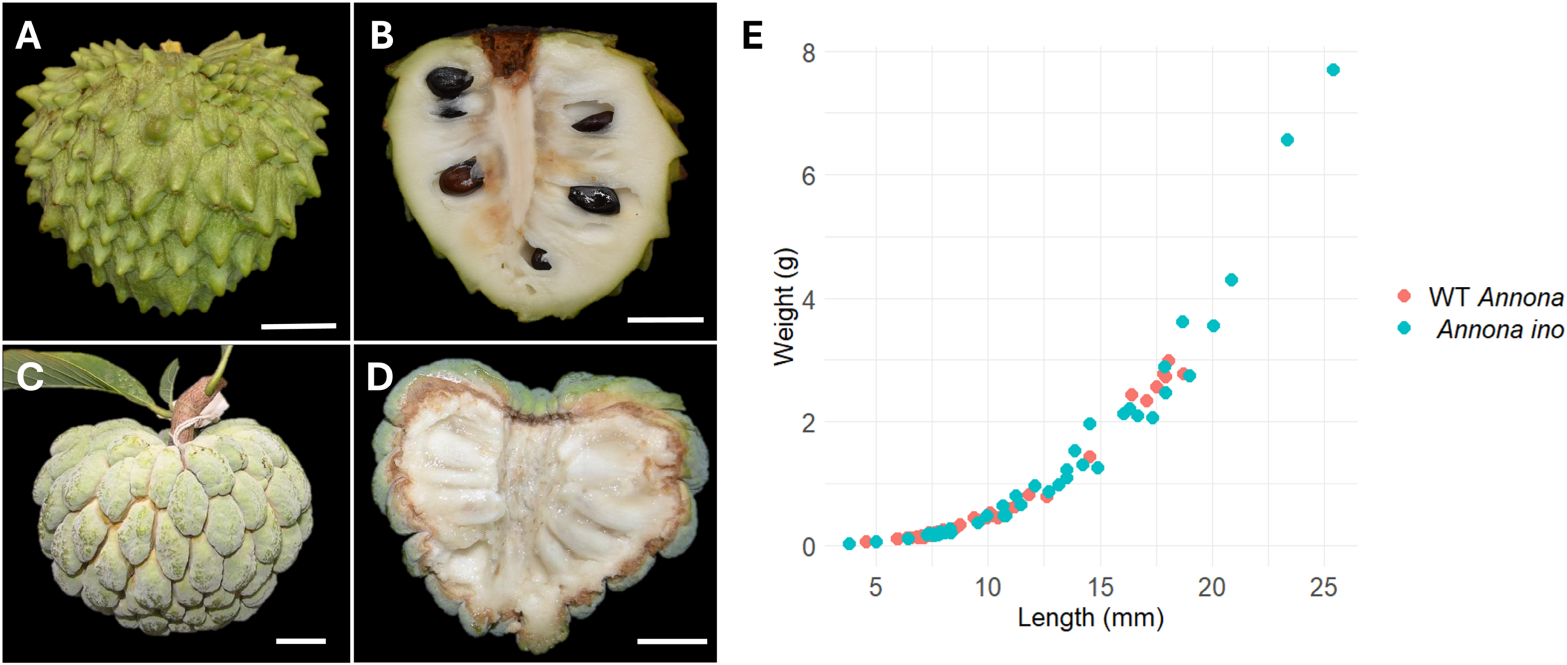
Fruit shape and size in wild‐type *Annona* and *Annona ino*. (A, B) Mature wild‐type *Annona* fruit. (C, D) Seedless mature fruit of *Annona ino*. (E) Fruit in *Annona ino* (blue dots) increased in length and weight in the same proportion as in wild‐type *Annona* (red dots) between 8 and 22 DAP. Scale bars: 2 cm.

**FIGURE 4:**
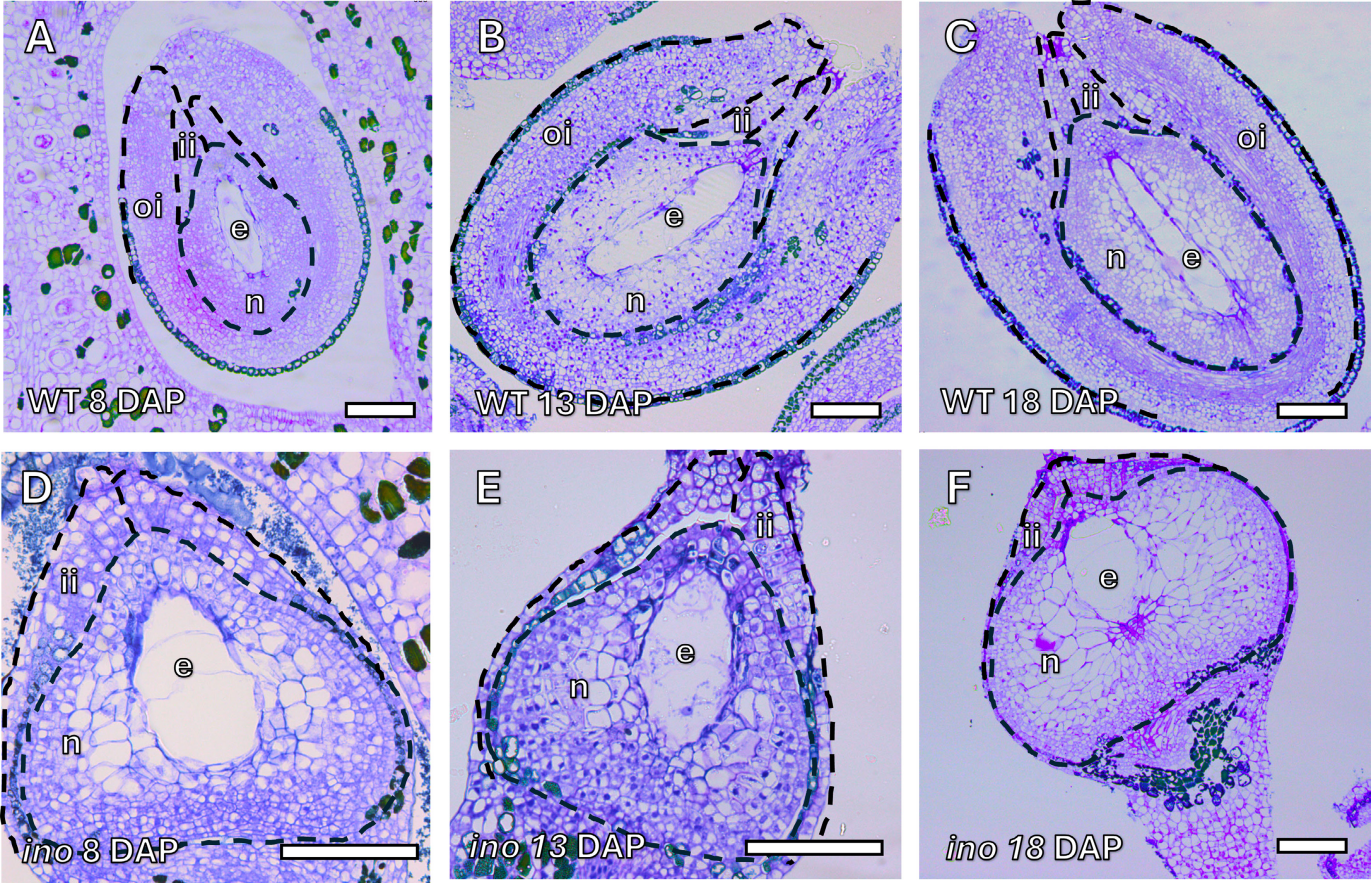
Shape of early stage developing seeds of wild‐type *Annona* and *Annona ino*. (A–C) The nucellus of immature wild‐type *Annona* seeds displayed an elliptical shape, narrowing at the micropylar region, and gradually elongating over days after pollination (DAP). The uniseriate endosperm appeared as a central bar‐like structure, surrounded by larger nucellar cells with prominent vacuoles. (D, E) In *Annona ino*, the nucellus maintained a more rounded shape. (F) At 18 DAP, the nucellus of *Annona ino* mutant enlarged, showing cells with prominent vacuoles. Sections (2 μm thick) were stained with periodic acid‐Schiff (PAS) reagent and toluidine blue. e, endosperm; ii, inner integument; n, nucellus; oi, outer integument. Scale bars: 100 μm.

Next, we evaluated fruit and seed development in both genotypes. The lack of the outer integument in *Annona ino* had critical consequences on seed development, leading to seed abortion and the production of seedless fruits. However, despite the lack of seeds, *Annona ino* fruits continued to develop normally, increasing in length and weight at the same rate as wild‐type *Annona* between 8 and 22 DAP, resulting in normally sized and shaped fruits (Figure 3). Differences in fruit skin, however, reflect the fact that wild‐type *Annona* ( *A. cherimola* ) and *Annona ino* ( *A. squamosa* ) are different species. Similarly, although fruit size increased slightly more rapidly in *Annona ino* between 18 and 22 DAP (Figure 3E), this could also be attributed to differences in fruit growth rate among different genotypes. Although overall fruit development proceeded normally over the full developmental period (120–146 days), the lack of the outer integument produced significant changes in early seed shape compared to wild‐type *Annona*. At 8 DAP, the nucellus of immature wild‐type *Annona* seeds exhibited an elliptical shape, narrowing at the micropylar region, and gradually elongated during the days following pollination (Figure 4A–C). This elongation was accompanied by the growth of a uniseriate endosperm, which appeared as a central bar‐like structure. During this process, the nucellus underwent cellular proliferation, with larger cells exhibiting prominent vacuoles adjacent to the endosperm. In contrast, in the *Annona ino* mutant, the nucellus maintained a more rounded shape, and this characteristic remained consistent even at 13 DAP (Figure 4D,E). By 18 DAP, the nucellus enlarged, showing cells with prominent vacuoles (Figure 4F).

**FIGURE 5:**
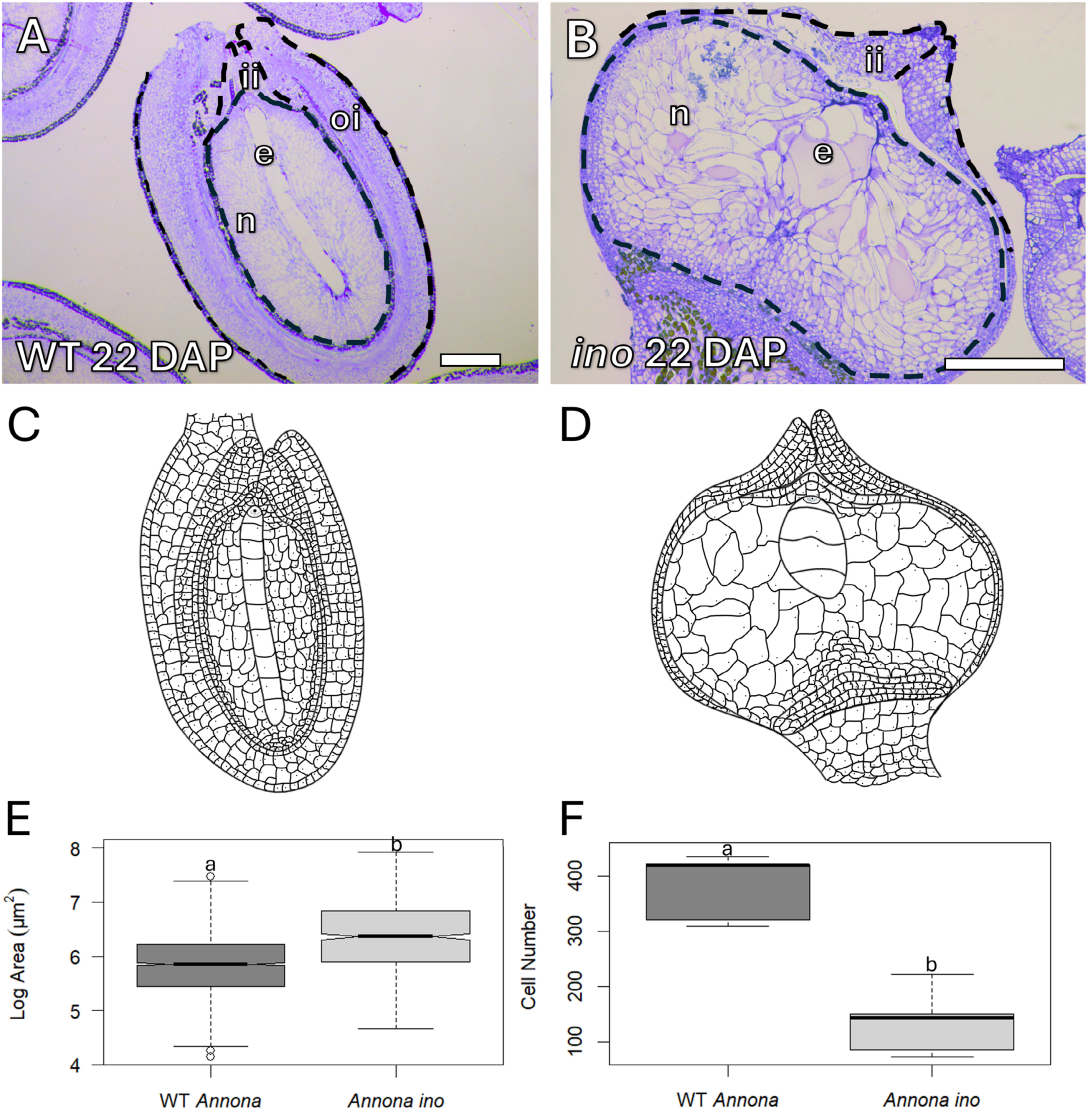
Differences in the nucellar cells between wild‐type *Annona* and *Annona ino* at 22 days after pollination (DAP). (A, C) In wild‐type *Annona*, the nucellus expanded toward the micropylar and chalazal domains, acquiring an elongated shape. The endosperm contained 9–10 cells. (B, D) In *Annona ino*, the nucellus expanded laterally, resulting in a more rounded and slightly swollen appearance at the poles. The endosperm showed a maximum of 4 cells and maintained a rounded structure. (E, F) In *Annona ino*, the nucellar cells surrounding the endosperm were significantly larger (E) and reduced in number compared to the wild‐type (F). Cell area data were analyzed using a Mann–Whitney test; cell number was compared using a Student's *t*‐test. Different letters above the log‐transformed cell area or cell number indicate a statistically significant difference. e, endosperm; ii, inner integument. n, nucellus; oi, outer integument. Scale bars: 200 μm.

By 22 DAP, the differences between the wild‐type *Annona* genotype and the *Annona ino* mutant became more pronounced. While in wild‐type *Annona* the nucellus grew toward both the micropylar and chalazal domains, acquiring an elongated shape, in the *Annona ino* mutant, growth occurred laterally, resulting in a more rounded and slightly flattened appearance at the poles. Furthermore, while in wild‐type *Annona* the uniseriate endosperm expanded to contain 9–10 cells (Figure 5A,C), in *Annona ino*, the endosperm remained reduced, with only 4–5 cells, maintaining a rounded structure (Figure 5B,D). The nucellar cell area surrounding the endosperm in *Annona ino* was significantly larger and contained more prominent vacuoles compared to wild‐type *Annona* (Figure 5E). While *Annona ino* exhibited an increase in the size of nucellar cells near the endosperm, it showed a decrease in the number of these cells compared to wild‐type *Annona* (Figure 5F). Based on single histological sections from five early seeds per genotype, the average number of nucellar cells in *Annona ino* was estimated to be 151 ± 53.0, whereas in wild‐type *Annona*, it was 380 ± 54.1. These values reflect measurements from representative sections and serve as an approximation of the overall nucellar cell number.

**FIGURE 6:**
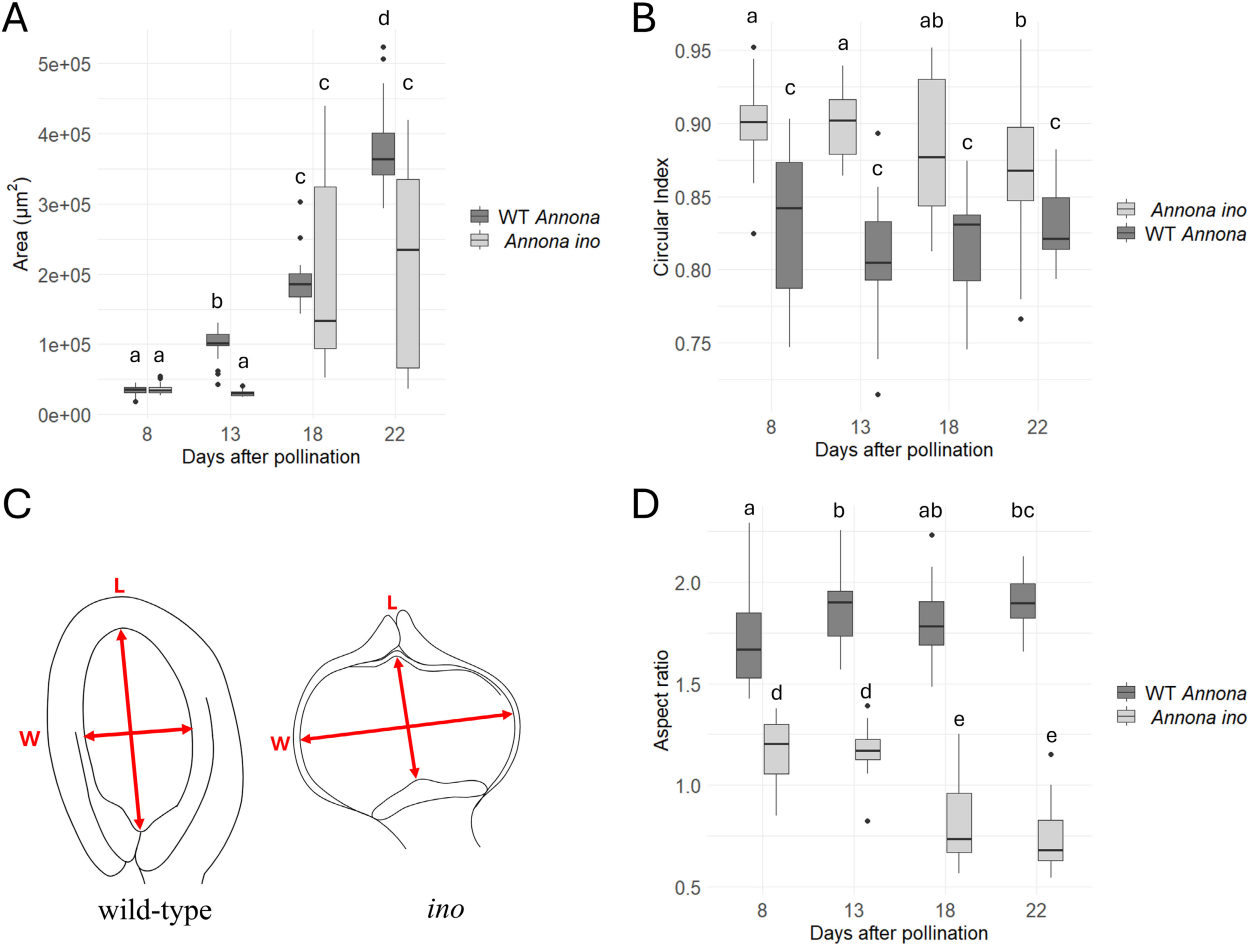
Consequences of the lack of the outer integument in seed shape in *Annona ino* compared to wild‐type *Annona*. (A) In wild‐type *Annona*, nucellus area increased uniformly between 8 and 22 days after pollination (DAP). In *Annona ino*, the area remained unchanged from 8 and 13 DAP before expanding at 18 and 22 DAP. (B) *Annona ino* showed higher values of circular index (CI) between 8 and 22 DAP, while wild‐type *Annona* exhibited lower values due to the polar proliferation of the nucellar cells. (C) Seed aspect ratio was defined as the ratio between length (L), and width (W) of the nucellus. (D) Wild‐type *Annona* exhibited higher AR values, consistent with polar growth, whereas *Annona ino* showed a decline at 18 and 22 DAP, indicating lateral expansion. Letters above the graph indicate statistically significant groups based on analysis of variance (ANOVA) followed by Tukey's test (*p* < 0.05).

In wild‐type *Annona*, the nucellus area increased gradually between 8 and 22 DAP, while in *Annona ino*, it remained unchanged from 8 to 13 DAP before expanding at 18 and 22 DAP (Figure 6A). The high variability at these later stages resulted from differences between ovules that continued developing and those that remained undifferentiated, resembling unpollinated ovules.

To further quantify these morphological differences, we calculated the circularity index (CI) and the AR. *Annona ino* consistently showed higher CI values (~0.9) between 8 and 22 DAP (Figure 6B), while wild‐type *Annona* exhibited lower values (0.80–0.85), reflecting its elongated shape due to nucellar cell divisions at the poles. In *Annona ino*, CI decreased at 18 and 22 DAP, reflecting lateral nucellus expansion due to the absence of the outer integument. Regarding the AR, which measures the length‐to‐width ratio of the nucellus (Figure 6C), wild‐type *Annona* exhibited higher AR values due to polar growth, while *Annona ino* showed a decline at 18 and 22 DAP, further confirming lateral expansion (Figure 6D). This expansion of the nucellus in *Annona ino* does not lead to further seed development. Although some mature *Annona ino* fruits occasionally contain remnants of aborted seeds, these likely originate from early seeds that initially developed an embryo and exhibited nucellar growth but failed to complete development. In the wild type, as reported in other related species such as *Asimina triloba* (Ferrer‐Blanco et al. 2025), the nucellus is displaced by the endosperm during development, and the mature fruit contains only endosperm as the nourishing tissue. These findings highlight the crucial role of the outer integument in enclosing the nucellus and preserving seed structure during embryo development.

### 3.3 Misregulation of Embryogenesis‐Related Genes in *Annona ino*

To assess the impact of outer integument on embryo development at the transcriptomic level, we compared gene expression profiles between *Annona ino* and wild‐type *Annona*. Total RNA was extracted from pistils containing the early‐developing seeds (PS) and pistils without the early‐developing seeds (P) at 4 and 8 DAP in both genotypes. Differentially expressed genes (DEGs) were then analyzed between PS and P samples from three biological replicates (Tables S1–S4). Additionally, we annotated DEGs with Gene Ontology (GO) terms and classified them into biological processes (Tables S5–S12).

**FIGURE 7:**
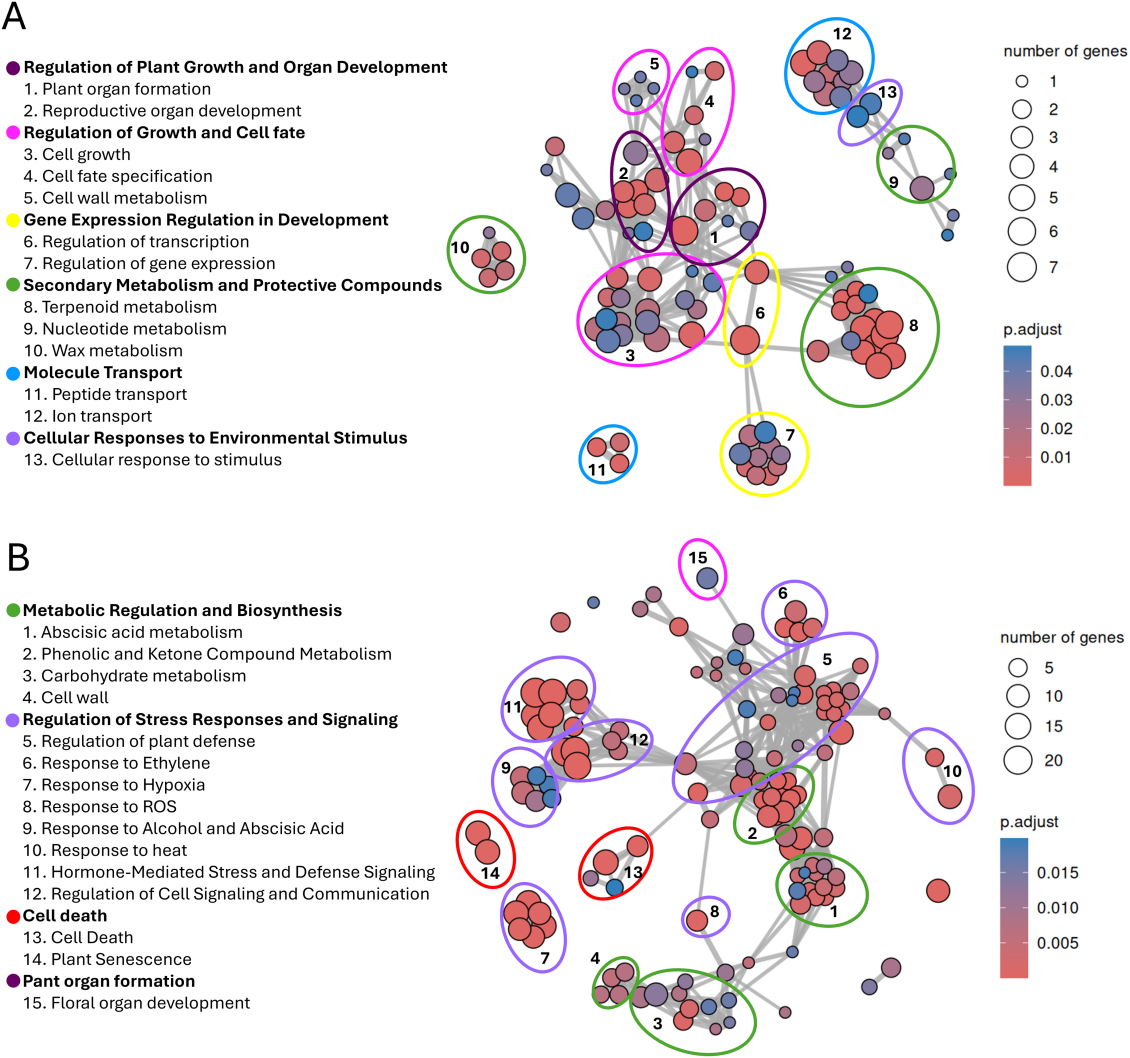
Functional enrichment analysis of differentially expressed genes (DEGs) between pistils with early‐developing seeds (PS) and pistils without early‐developing seeds (P) samples in wild‐type *Annona* and *Annona ino* at 4 days after pollination (DAP). (A) In wild‐type *Annona*, enriched Gene Ontology (GO) terms were grouped into functional clusters (represented by colored ellipses) associated with reproductive structure development, embryo, seed, and fruit development. (B) In *Annona ino*, enriched GO cluster were primarily related to metabolic regulation and biosynthesis, regulation of stress responses and signaling, cell death and plant organ formation. The number of enriched genes in each GO term is indicated by the size of the dots, and the color intensity represents the range of adjusted *p* values (p. adjust).

In wild‐type *Annona*, at 4 DAP, 142 genes were differentially expressed between PS and P samples, with 105 upregulated and 37 downregulated in the samples containing the post‐pollination ovules (Table S1). The enriched GO terms were linked within a network and grouped into distinct clusters. The GO enrichment analysis revealed that PS samples showed an overrepresentation of genes associated with the biological processes of reproductive structure, embryo, seed, and fruit development. The main functional clusters were related to the plant growth regulation, organ development, cell growth and fate determination, gene expression in development, secondary metabolism and protective compounds, molecule transport, and cellular responses to stimuli (Figure 7A). These results suggest enhanced metabolic activity in wild‐type *Annona* developing ovules associated with cell development and specification, essential for the growth and differentiation of the embryo and all the tissues that will form the seed. Within the biological process GO category, the most highly represented terms were floral organ development, terpenoid metabolic, and inorganic ion transport (Tables S5 and S9).

In *Annona ino*, at 4 DAP, 481 genes were differentially expressed between PS and P samples, with 388 upregulated and 93 downregulated in the samples containing the post‐pollination ovules (Table S2). The enrichment GO terms grouped into clusters related to metabolic regulation and biosynthesis, regulation of stress responses and signaling, cell death, and plant organ formation (Figure 7B). In the metabolic regulation and biosynthesis cluster, we found enrichment in GO terms associated with abscisic acid (ABA) metabolism. The most highly represented GO terms in the biological process category included cell death and responses to salicylic acid (SA), jasmonic acid (JA), and ethylene (Tables S6 and S10). The enrichment analysis of DEGs at 4 DAP showed that gene expression in *Annona ino* ovules is focused on cell death and stress responses, whereas in wild‐type *Annona* ovules, the expression profile is geared toward cell growth and differentiation. These results suggest that normal embryo development occurred in wild‐type *Annona* due to a high representation of genes related to plant and reproductive organ development in the ovule. In contrast, these genes were absent among the most enriched terms in *Annona ino* ovules, suggesting that the mutant fails to generate the necessary signals to ensure the correct development of the embryo structures.

At 8 DAP, a total of 106 genes were differentially expressed between PS and P samples in wild‐type *Annona* (Table S3), compared to 673 differentially expressed genes observed in *Annona ino* (Table S4). The enriched GO terms in PS samples compared to P samples, in wild‐type *Annona* early‐developing seeds, were grouped into similar clusters to those observed at 4 DAP (Figure S2A). The ontology analysis revealed an abundance of transcripts related to plant organ formation, cell growth and differentiation, and molecular transport, all of which are associated with embryo and seed development (Tables S7 and S11). Furthermore, the enriched GO terms in *Annona ino* at 8 DAP in PS samples were consistent with those observed at 4 DAP (Figure S2B; Tables S8 and S12). These results indicate that while post‐pollination ovules in wild‐type *Annona* continue to develop as expected at 8 DAP, post‐pollination ovules in *Annona ino* fail to progress toward the formation of a functional embryo.

Supporting this, a comparative analysis of DEGs between *Annona ino* and wild‐type *Annona* PS samples revealed differential expression of key genes previously implicated in embryogenesis and seed formation. However, it is important to note that, due to the limited size of the embryo and endosperm at this stage, and their embedding within a large number of maternal sporophytic cells, the DEGs identified are more likely to reflect changes in the nucellus and the absence of the outer integument, rather than direct transcriptional signatures of embryogenesis. We have therefore interpreted the RNA‐seq data with this limitation in mind.

At 4 DAP, wild‐type samples showed significantly higher expression levels of orthologs of *INO*, *PENTATRICOPEPTIDE REPEAT 4* (*PPR4*), and *PINFORMED 1* (*PIN1*), all of which are known to play critical roles in ovule patterning and embryo polarity (Figure S3). As expected, no expression of the *INO* gene was detected in *Annona ino*. In addition, several putative orthologous genes previously linked to embryo‐defective and seed‐related phenotypes in *Arabidopsis* mutants (Meinke et al. 2008) showed reduced expression in *Annona ino* early‐developing seeds at 4 DAP compared to wild‐type *Annona* (Figure S4A). Interestingly, some of these genes were overexpressed in *Annona ino* post‐pollination ovules (Figure S4B), suggesting a possible compensatory response or dysregulation linked to the abnormal development trajectory. Together, these data reinforce that the absence of the outer integument in *Annona ino* alters the transcriptional landscape of the ovule, disrupting the precise gene expression dynamics required for successful embryo initiation and seed development.

## 4 Discussion

In the *Arabidopsis ino* mutant, the absence of the outer integument inhibits embryo sac development, preventing ovules from attracting pollen tubes and leading to ovule degeneration (Skinner and Gasser 2009). In contrast, in the *Annona ino* mutant, the embryo sac develops fully, pollen tube growth is normal, with 41% of pollen tubes reaching the nucelli (Lora et al. 2011, 2019), and fertilization occurs successfully, although only 27.3% of the early seeds progress to the two‐cell embryo stage. However, fertilization in the *Annona ino* mutant does not result in seed formation (Lora et al. 2011). Additionally, while the endosperm begins to develop into a 3–4 cell structure, it fails to continue dividing. These results suggest that fertilization in *Annona* can occur in the absence of the outer integument, but this structure seems to be essential to support subsequent embryo and endosperm development.

In seed plants, maternal tissues closely associated with the embryo play a key role in embryogenesis by facilitating nutrient transfer and transmitting essential signals for embryo development (Garcia et al. 2005; Burkart‐Waco et al. 2013; Yang et al. 2023). In *Arabidopsis*, sugars are transferred from the outer to the inner integument, and possibly to the suspensor, followed by their release into the seed apoplasm membrane transport systems (Stadler et al. 2005). Sucrose transporters are crucial for supplying nutrients from maternal tissues (seed coat and endosperm) to the developing embryo (Chen et al. 2015). During early seed development, the integuments play an important role in supporting embryo formation by supplying auxin. After fertilization, auxin levels rise in the ovule due to upregulated auxin biosynthesis in the integuments, and this maternal auxin is essential for proper embryo development (Robert et al. 2018).

Therefore, the failure of seed development in *Annona* ino could result not only from the absence of biochemical signals from the outer integument but also from the loss of its physical support, which may alter the fate or structure of internal tissues such as the nucellus. How the resulting “bloated” nucellus contributes to seed abortion remains unclear, but it may involve changes in cell identity or developmental trajectory in the absence of the outer integument.

### 4.1 Divergent Ovule Gene Expression: Fertilization in Wild‐Type *Annona* Versus Cell Death in *Annona ino*

Beyond the organ and cell level, gene expression profiles of wild‐type *Annona* and *Annona ino* ovules differed during the early stages of seed development. In wild‐type *Annona*, the most highly represented GO term in the enrichment analysis was related to terpenoid metabolism and inorganic ion transport. The overexpressed genes involved in the terpenoid metabolic process were associated with gibberellin (GA) metabolism. Several phytohormones, including GA, are closely correlated with endosperm proliferation and embryo growth (Sun et al. 2010). In *Arabidopsis*, fertilization induces auxin accumulation in the ovule, which rapidly upregulates GA biosynthesis genes, resulting in enhanced GA production specifically within developing seeds (Dorcey et al. 2009). Additionally, the upregulated genes related to the ion transport term in wild‐type *Annona* are mostly involved in Ca^2+^ transport, which is also an indicator of effective fertilization. In both plants and animals, evidence suggests that fertilization initiates the first embryonic events by opening calcium channels, leading to a Ca^2+^ influx followed by Ca^2+^oscillations and accumulation, which may subsequently trigger additional aspects of egg activation (Antoine et al. 2001). Thus, the enrichment of GO terms associated with GA metabolism and Ca^2+^ transport in wild‐type *Annona* post‐pollination ovules at 4 DAP strongly suggests that fertilization has occurred.

In contrast, although the histological analysis indicated apparent fertilization in *Annona ino*, its ovules at 4 DAP showed a predominance of GO terms related to SA, JA, and ethylene responses, which are known to be key regulators in plant programmed cell death (PCD) pathways (Burkart‐Waco et al. 2013; Wang et al. 2021). SA is known to regulate seed germination, embryo development, and senescence, and high SA levels have been linked to seed abortion in seedless grapes (Royo et al. 2018). JA regulates various aspects of plant growth and development, including embryonic axis elongation, flowering, leaf senescence, root formation, and stress tolerance (He et al. 2002; Hu et al. 2017; Huang et al. 2017). The implication of JA signaling in leaf senescence may suggest its role in ovule senescence, considering the evolutionary origin of ovules from ancestral foliar organs. In addition, ethylene, the most studied phytohormone associated with PCD (Trobacher 2009), is activated during fertilization in *Arabidopsis* and mediates the elimination of the synergid cells (Völz et al. 2013; Maruyama et al. 2015). The correlation between JA, GA, and ethylene with PCD is further supported by a study in seedless pears, where elevated levels of these phytohormones were observed. Moreover, the application of JA and SA in wild‐type pear genotypes induced ovule PCD (Wang et al. 2021). Additionally, genes related to SA biosynthesis were upregulated in aborted ovules of *Castanea henryi* (Qiu et al. 2023). Nevertheless, an analysis of the early embryo *Arabidopsis* transcriptome revealed enrichment in genes related to biotic stress, such as abscisic acid (ABA), JA, and SA responsive genes. These results indicate that while gene expression in wild‐type *Annona* ovules was directed toward fertilization, embryo development, and seed formation, *Annona ino* ovules primarily expressed genes related to stress responses and PCD, failing in the generation of essential signals for embryo and endosperm development.

Transcriptome analyses revealed that the absence of the outer integument in *Annona ino* affected key genes related to embryo and seed development. Higher expression levels of the *Annona* orthologs, such as *INO*, *PPR4*, and *PIN1*, were observed in wild‐type *Annona* post‐pollination ovules at 4 DAP. These genes have been implicated in processes associated with embryo development (Aida et al. 2002; Li et al. 2021; Sun et al. 2021), and their reduced expression levels in *Annona ino* could be linked to the subsequent failure in embryo formation. Previous studies in *Arabidopsis* have shown that *INO* mRNA is most abundant during early seed development and gradually decreases as seed development progresses (Sun et al. 2021). Furthermore, the loss of function of PPR proteins typically results in defects in embryogenesis and endosperm development (Barkan and Small 2014; Li et al. 2021). Additionally, the observed differences in PIN1 expression between genotypes could be related to the activity of PIN‐family auxin transporters in the ovule integuments. Sporophytic tissues play a key role in early seed development by supplying auxin from the maternal integuments to the developing embryo (Robert et al. 2018). In *Arabidopsis* and maize, maternally produced auxin is essential for embryo patterning, and auxin levels increase significantly in ovule tissues after pollination due to upregulated biosynthesis in the integuments. In *Annona ino*, the absence of the outer integument may result in reduced auxin levels after fertilization, potentially contributing to seed abortion.

Thus, the reduction of the expression of these essential genes for embryo and endosperm development in *Annona ino* enhances the critical role of the outer integument in mediating gene expression and ensures the correct proliferation of the seed structures.

### 4.2 The Role of the Outer Integument in Ovule Morphology

The absence of the outer integument in *Annona ino* not only alters gene expression contributing to the observed transcriptional shifts but also impacts ovule morphology, ultimately influencing seed development and viability. Most flowering plants exhibit anatropous bitegmic ovules, establishing a relationship between ovule curvature and the presence of two integuments (Endress 2011). However, some species, such as those in the genus *Impatiens* and certain *Prunus* species, possess a single integument (McAbee et al. 2005; Lora et al. 2015); in these cases, it has been reported that the single integument results from the fusion of both integuments during ovule development while still relying on the role of the *INO* gene in ovule curvature (Lora et al. 2015). The occurrence of both a single integument and an orthotropous ovule is rare among angiosperms and can be found in the families Ceratophyllaceae, Hydnoraceae, and Piperaceae (genus *Peperomia*), although there are no genetic studies addressing the genetic regulatory mechanisms involved in their single integument development or the potential absence of *INO* expression (Igersheim and Endress 1998). This contrasts with gymnosperms, which typically have orthotropous ovules, with the exception of the Gnetales (Frohlich and Chase 2007; Rudall 2021), and no orthologs of *INO* have been identified in gymnosperms (Bartholmes et al. 2012). This highlights the unique nature of the outer integument and the *INO* gene as distinctive features of flowering plants.

INO belongs to the YABBY family of transcription factors, which are involved in establishing abaxial–adaxial polarity in plant organs (Siegfried et al. 1999; Villanueva et al. 1999). The *YABBY* gene family has undergone several duplication events prior to the diversification of angiosperms, and since *INO* appears to be specific to angiosperms (Bartholmes et al. 2012), this suggests that the evolution of *INO* could be closely linked to the emergence of bitegmic ovules, a defining trait of angiosperms (Villanueva et al. 1999; Yamada et al. 2003). While the innovative role of *INO* in ovule curvature in angiosperms is well established, its function may extend beyond curvature, potentially influencing inner tissues such as the nucellus. However, few studies have directly explored this possibility.

In *Arabidopsis*, seed development occurs through two distinct stages: first, the early anisotropic growth phase linked to the division and directional growth of the outer integument in which the seed primarily elongates rather than expanding in width or thickness; this is followed by a slower isotropic phase that involves uniform growth of the cells in the outer integument (Bauer et al. 2024). In *Annona ino*, seeds exhibit a more rounded nucellus compared to the bitegmic wild‐type *Annona*, due to the absence of the anisotropic growth driven by the outer integument. This morphological characteristic has also been observed in other species of early‐divergent angiosperms, such as those in the genus *Peperomia* in the Piperales, which, like the Annonaceae, belong to the Eumagnoliid clade and exhibit orthotropous unitegmic ovules (Johnson 1900). The shape of the fertilized ovules, in which the zygote is visible, resembles the circular disposition observed in *Annona ino* ovules (Madrid and Friedman 2010). Additionally, in *Peperomia*, the endosperm is not uniseriate and is disposed surrounding the zygote (Johnson 1900). These similarities in ovule morphology between *Peperomia* and *Annona ino* underscore the crucial role of the outer integument in maintaining ovule structure and proper development. The maternal sporophytic control of endosperm and seed growth has also been demonstrated in *Arabidopsis ttg2* mutants. In these mutants, the deletion of the *TGG* function causes the seed coat to become more rigid than in the wild type, resulting in a more rounded seed shape and reduced endosperm and embryo development (Garcia et al. 2005).

## 5 Conclusion

Our study demonstrates that while fertilization can occur in the *Annona ino* mutant, the absence of the outer integument prevents successful seed formation and disrupts both embryo and endosperm development. Gene expression analysis revealed key differences between wild‐type *Annona* and *Annona ino* post‐pollination ovules. In wild‐type *Annona*, the gene expression profile is strongly associated with fertilization, embryo development, and seed formation, while *Annona ino* ovules predominantly exhibit gene expression patterns related to PCD and stress responses. Beyond its impact on gene expression, the absence of the outer integument in *Annona ino* also affects ovule morphology, highlighting the critical role of the outer integument in regulating seed development. The unique properties of the outer integument and its involvement in ovule curvature, as well as its role in nutrient transfer and signaling, emphasize the complex maternal influence on seed development in flowering plants. Overall, these findings contribute to our understanding of the essential role of the outer integument in embryo and seed formation, offering insights into the broader evolutionary significance of integument evolution in angiosperms.

## Author Contributions

M.G.‐L., J.I.H., and J.L. planned and designed the research. M.G.‐L. performed most of the experiments, except for the transcriptome analysis, which was conducted by E.L.‐G. and N.F.‐P. All the authors discussed the results and contributed to the preparation of the final manuscript.

## Conflicts of Interest

The authors declare no conflicts of interest.

## Supporting information


**Figure S1:** Adjacent serial sections of embryos in wild‐type *Annona*. (A, B) Adjacent serial sections showing the first cellular division forming a two‐celled embryo at 13 DAP and (C, D) 22 DAP in wild‐type *Annona*. emb, embryo. Scale bars: 25 μm.
**Figure S2:** Functional enrichment analysis of differentially expressed genes (DEGs) between pistils with early‐developing seeds (PS) and pistils without early‐developing seeds (P) samples in wild‐type *Annona* and *Annona ino* at 8 days after pollination (DAP). (A) In wild‐type *Annona*, enriched Gene Ontology (GO) terms were grouped into functional clusters (represented by elliptical colored forms) associated with plant organ formation, cell growth and differentiation, and molecular transport, all of which are associated with ovule development and seed formation. (B) In *Annona ino*, enriched GO clusters were primarily related to metabolic regulation and biosynthesis, regulation of stress responses and signaling, cell death and growth and development regulation. The number of enriched genes in each GO term is indicated by the size of the dots, and the color intensity represents the range of adjusted *p* values (p. adjust).
**Figure S3:** Differentially expressed genes (DEGs) involved in embryogenesis between wild‐type *Annona* and *Annona ino* pistils containing early‐developing seeds (PS) at 4 days after pollination (DAP). Each bar indicates standard error in three biological replicates (*p ≤ 0.05).
**Figure S4:** Differential expressed genes (DEGs) of transcripts for embryogenesis related putative genes in early‐stage fruits at 4 days after pollination (DAP) of wild‐type *Annona* and *Annona ino*. The orthologous genes give a defective embryo and/or seed phenotype in *Arabidopsis* mutants. On the *Y*‐axis, the embryo‐defective (EMB) mutant of *Arabidopsis* is followed by the gene ID of *Annona cherimola* . (A) Most of the embryo defective genes exhibited reduced expression in *Annona ino* early seeds at four DAP compared to wild‐type *Annona*. (B) However, some of these genes were overexpressed in *Annona ino* ovules.

**Table S1:** Differentially expressed genes (DEGs) in pistils with early‐developing seeds (PS) versus pistils without early‐developing seeds (P) in wild‐type *Annona* at 4 days after pollination (DAP).
**Table S2:** Differentially expressed genes (DEGs) in pistils with early‐developing seeds (PS) versus pistils without early‐developing seeds (P) in *Annona ino* at 4 days after pollination (DAP).
**Table S3:** Differentially expressed genes (DEGs) in pistils with early‐developing seeds (PS) versus pistils without early‐developing seeds (P) in wild‐type *Annona* at 8 days after pollination (DAP).
**Table S4:** Differentially expressed genes (DEGs) in pistils with early‐developing seeds (PS) versus pistils without early‐developing seeds (P) in *Annona ino* at 8 days after pollination (DAP).

**Table S5:** Enriched genes in pistils with early‐developing seeds (PS) versus pistils without early‐developing seeds (P) in wild‐type *Annona* at 4 days after pollination (DAP).
**Table S6:** Enriched genes in pistils with early‐developing seeds (PS) versus pistils without early‐developing seeds (P) in *Annona ino* at 4 days after pollination (DAP).
**Table S7:** Enriched genes in pistils with early‐developing seeds (PS) versus pistils without early‐developing seeds (P) in wild‐type *Annona* at 8 days after pollination (DAP).
**Table S8:** Enriched genes in pistils with early‐developing seeds (PS) versus pistils without early‐developing seeds (P) in *Annona ino* at 8 days after pollination (DAP).
**Table S9:** Functional enrichment analysis of differentially expressed genes (DEGs) between pistils with early‐developing seeds (PS) and pistils without early‐developing seeds (P) samples in wild‐type *Annona* at 4 days after pollination (DAP).
**Table S10:** Functional enrichment analysis of differentially expressed genes (DEGs) between pistils with early‐developing seeds (PS) and pistils without early‐developing seeds (P) samples in *Annona ino* at 4 days after pollination (DAP).
**Table S11:** Functional enrichment analysis of differentially expressed genes (DEGs) between pistils with early‐developing seeds (PS) and pistils without early‐developing seeds (P) samples in wild‐type *Annona* at 8 days after pollination (DAP).
**Table S12:** Functional enrichment analysis of differentially expressed genes (DEGs) between pistils with early‐developing seeds (PS) and pistils without early‐developing seeds (P) samples in *Annona ino* at 8 days after pollination (DAP).

## Data Availability

The sequencing data generated in this study has been deposited in the NCBI Sequence Read Archive (SRA) under BioProject ID PRJEB97455.
