# Supplementary material for: Beyond Structure: The Role of the Outer Integument in Embryo, Endosperm, and Seed Development in Annona
Source: Physiol Plant. 2025 Sep 24;177(5):e70538. doi: 10.1111/ppl.70538 (PMC12460975; doi:10.1111/ppl.70538)
Supplement: Supplementary file 1 — Figure S1: Adjacent serial sections of embryos in wild‐type Annona. (A, B) Adjacent serial sections showing the first cellular division forming a two‐celled embryo at 13 DAP and (C, D) 22 DAP in wild‐type Annona. emb, embryo. Scale bars: 25 μm. Figure S2: Functional enrichment analysis of differentially expressed genes (DEGs) between pistils with early‐developing seeds (PS) and pistils without early‐developing seeds (P) samples in wild‐type Annona and Annona ino at 8 days after pollination (DAP). (A) In wild‐type Annona, enriched Gene Ontology (GO) terms were grouped into functional clusters (represented by elliptical colored forms) associated with plant organ formation, cell growth and differentiation, and molecular transport, all of which are associated with ovule development and seed formation. (B) In Annona ino, enriched GO clusters were primarily related to metabolic regulation and biosynthesis, regulation of stress responses and signaling, cell death and growth and development regulation. The number of enriched genes in each GO term is indicated by the size of the dots, and the color intensity represents the range of adjusted p values (p. adjust). Figure S3: Differentially expressed genes (DEGs) involved in embryogenesis between wild‐type Annona and Annona ino pistils containing early‐developing seeds (PS) at 4 days after pollination (DAP). Each bar indicates standard error in three biological replicates (*p ≤ 0.05). Figure S4: Differential expressed genes (DEGs) of transcripts for embryogenesis related putative genes in early‐stage fruits at 4 days after pollination (DAP) of wild‐type Annona and Annona ino. The orthologous genes give a defective embryo and/or seed phenotype in Arabidopsis mutants. On the Y‐axis, the embryo‐defective (EMB) mutant of Arabidopsis is followed by the gene ID of Annona cherimola . (A) Most of the embryo defective genes exhibited reduced expression in Annona ino early seeds at four DAP compared to wild‐type Annona. (B) However, some [file PPL-177-e70538-s003.pdf]

Beyond Structure: The Role of the Outer Integument in Embryo, Endosperm and Seed Development in *Annona*

María Garcia-Lezama, José I. Hormaza, Enrique Lopez-Gomez, Noé Fernandez-Pozo, Jorge Lora

Figures S1-S4

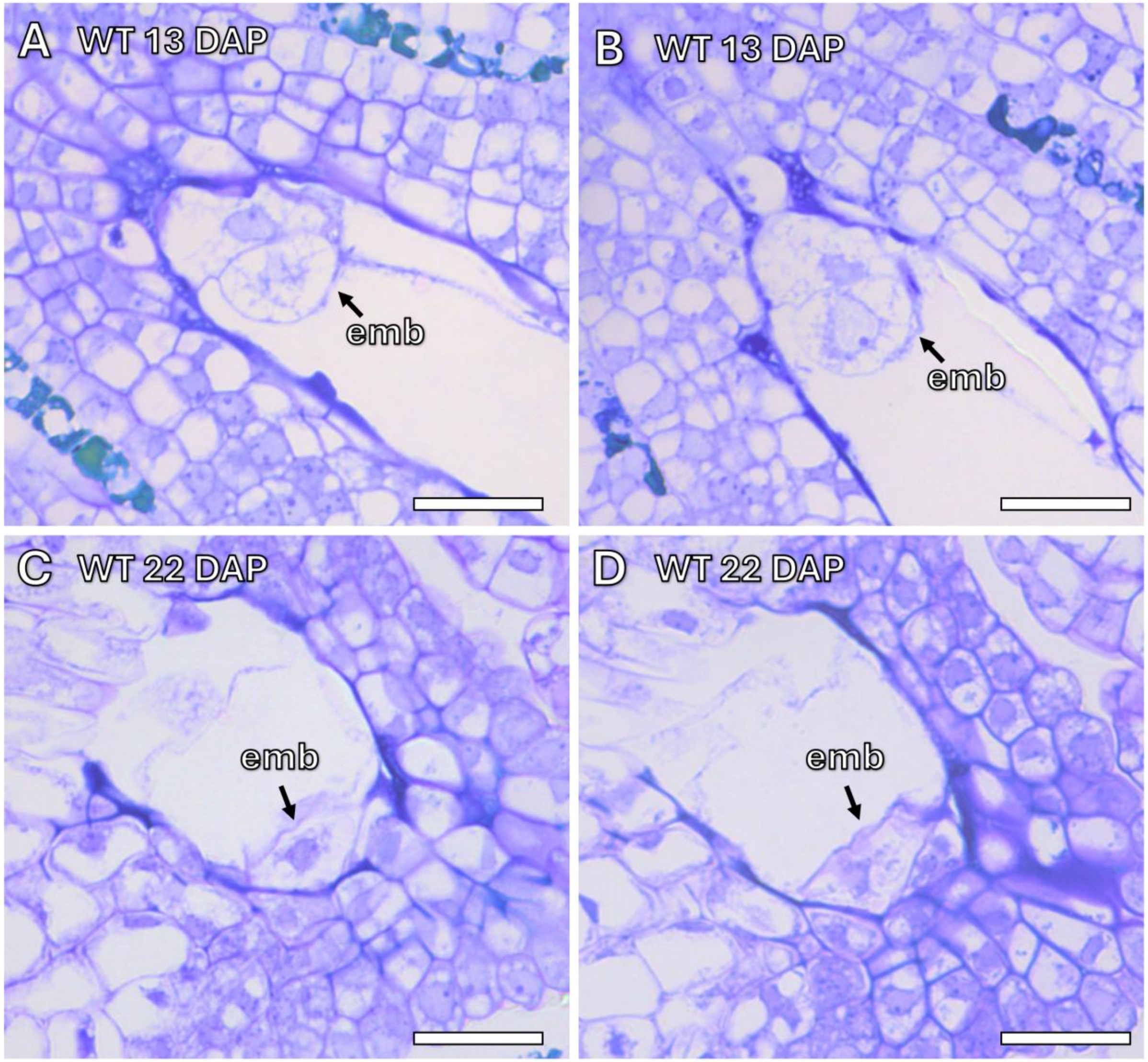

FIGURE S1. Adjacent serial sections of embryos in wild-type *Annona*. (A, B) Adjacent serial sections showing the first cellular division forming a two-celled embryo at 13 DAP and (C, D) 22 DAP in wild-type *Annona*. emb, embryo. Scale bars: 25 µm.

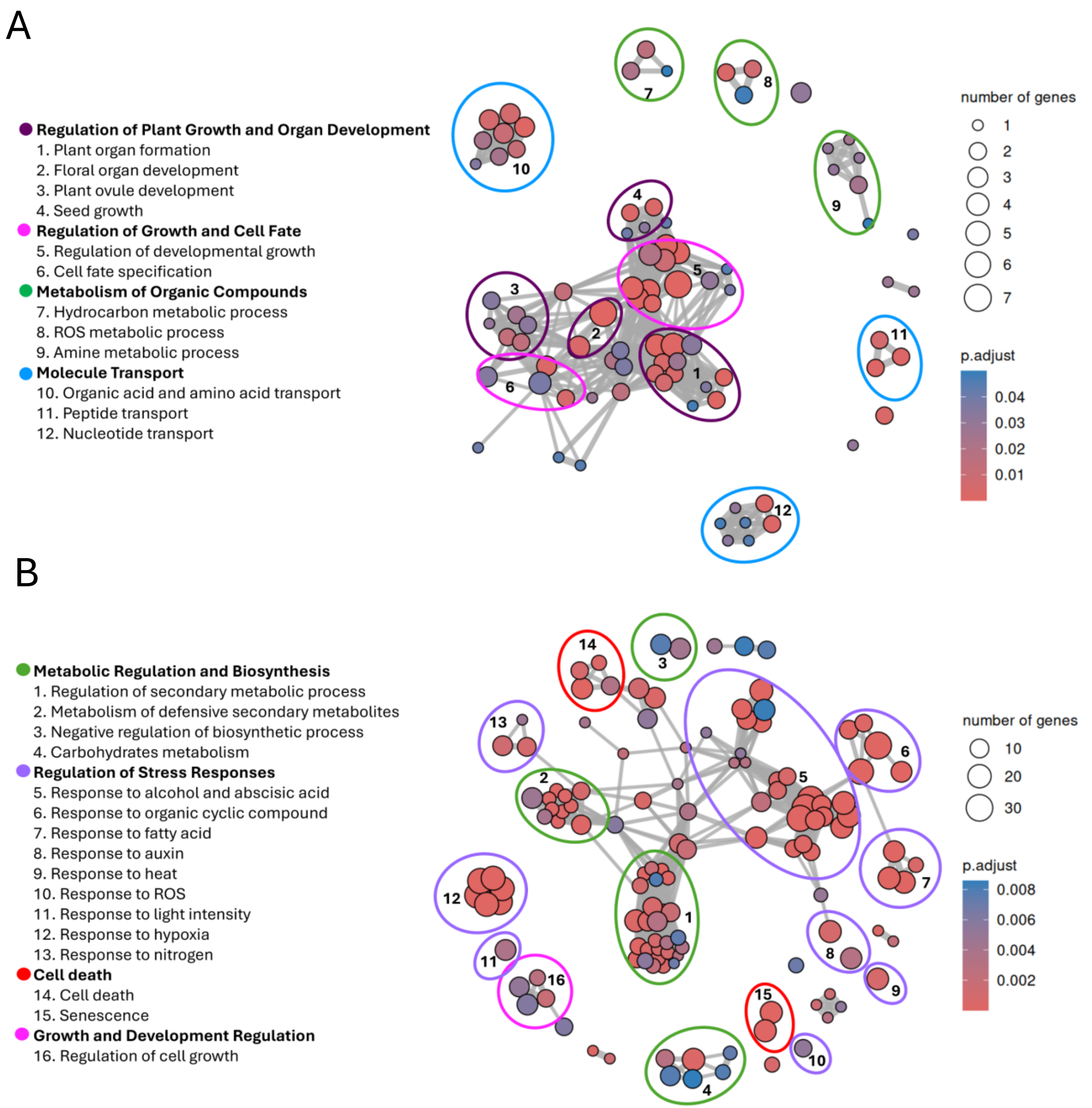

FIGURE S2. Functional enrichment analysis of differentially expressed genes (DEGs) between pistils with early-developing seeds (PS) and pistils without early-developing seeds (P) samples in wild-type *Annona* and *Annona ino* at 8 days after pollination (DAP). (A) In wild-type *Annona*, enriched Gene Ontology (GO) terms were grouped into functional clusters (represented by elliptical colored forms) associated with plant organ formation, cell growth and differentiation, and molecular transport, all of which are associated with ovule development and seed formation. (B) In *Annona ino*, enriched GO clusters were primarily related to metabolic regulation and biosynthesis, regulation of stress responses and signaling, cell death and growth and development regulation. The number of enriched genes in each GO term is indicated by the size of the dots, and the color intensity represents the range of adjusted *p* values (p. adjust).

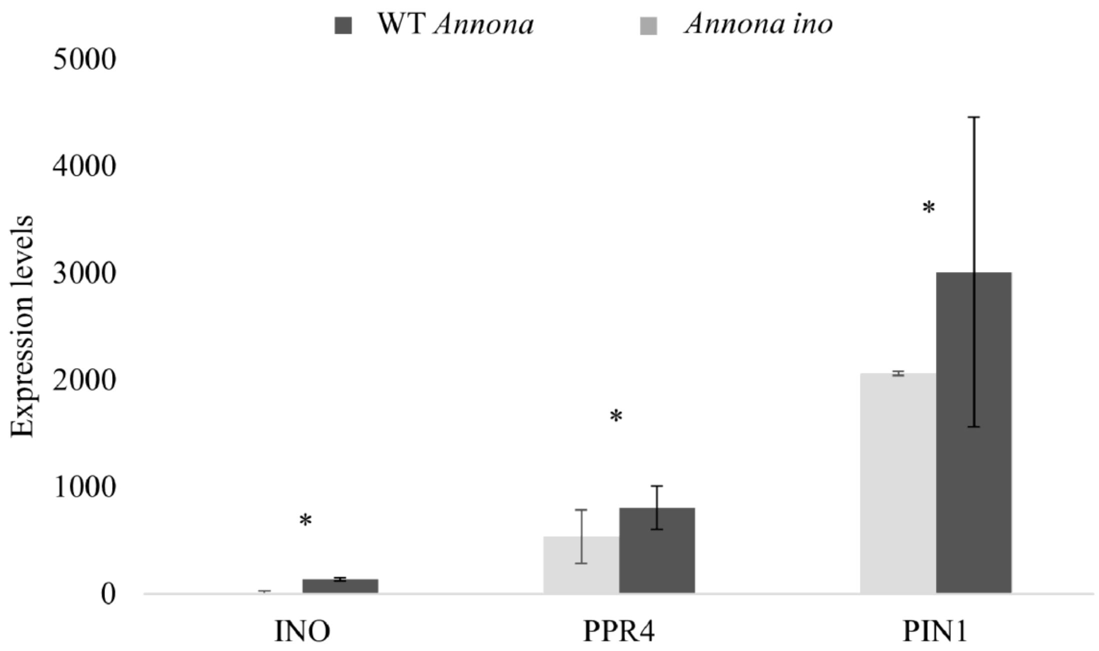

FIGURE S3. Differentially expressed genes (DEGs) involved in embryogenesis between wild-type *Annona* and *Annona ino* pistils containing early-developing seeds (PS) at 4 days after pollination (DAP). Each bar indicates standard error in three biological replicates (*$p \leq 0.05$).

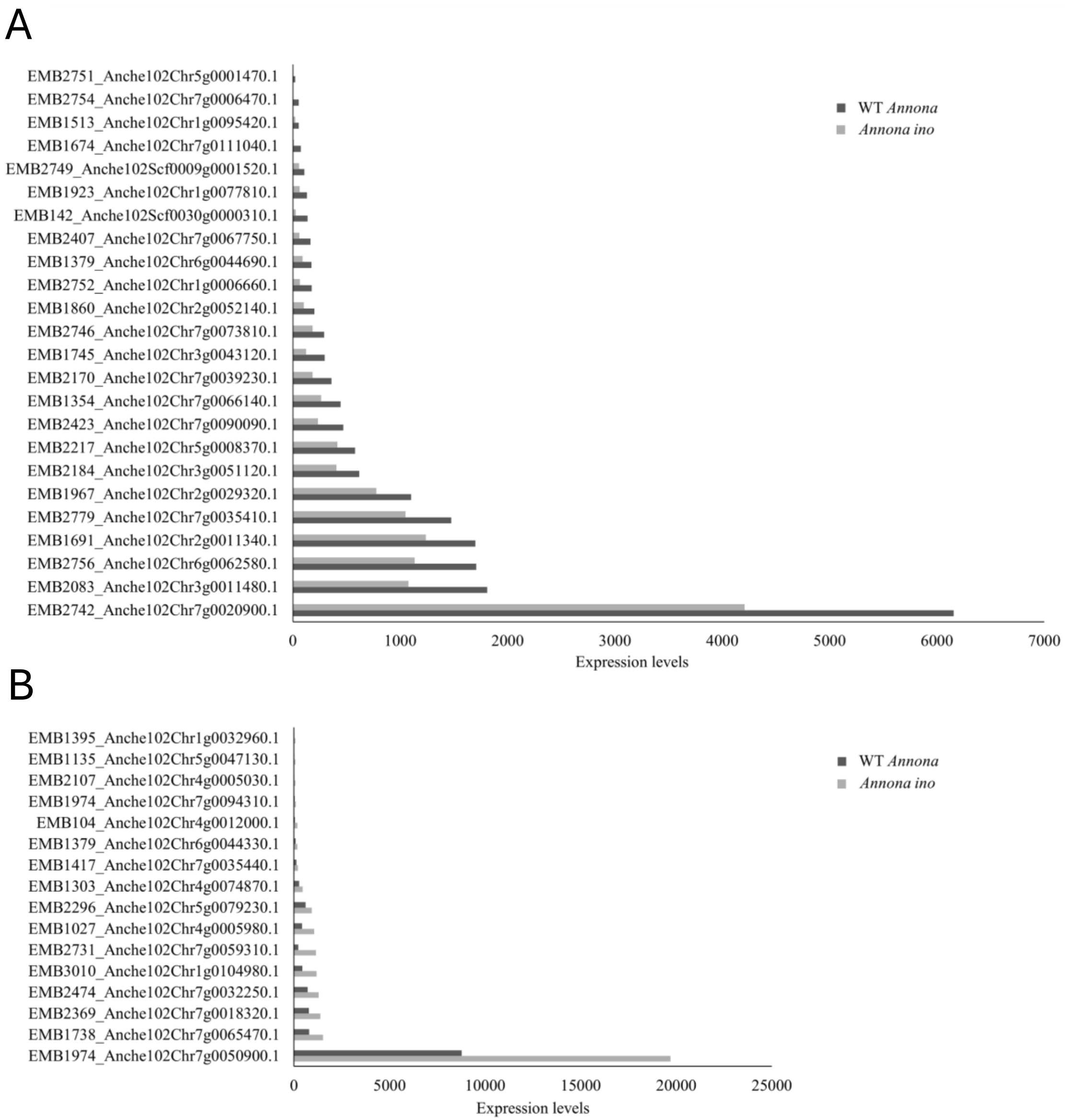

FIGURE S4. Differential expressed genes (DEGs) of transcripts for embryogenesis related putative genes in early-stage fruits at 4 days after pollination (DAP) of wild-type *Annona* and *Annona ino*. The orthologous genes give a defective embryo and/or seed phenotype in *Arabidopsis* mutants. On the Y-axis, the embryo-defective (EMB) mutant of *Arabidopsis* is followed by the gene ID of *Annona cherimola*. (A) Most of the embryo defective genes exhibited reduced expression in *Annona ino* early seeds at 4 DAP compared to wild-type *Annona*. (B) However, some of these genes were overexpressed in *Annona ino* ovules.
